# A review: research progress on intelligent technologies for orchard yield monitoring

**DOI:** 10.3389/fpls.2026.1879872

**Published:** 2026-06-30

**Authors:** Shengyi Zhao, Han Lin, Yong Jiang, Jizhan Liu

**Affiliations:** 1 School of Agricultural Engineering, Jiangsu University, Zhenjiang, China; 2 National Digital Agricultural Equipment (AI and Agricultural Robotics) Innovation Sub-Centre, Jiangsu University, Zhenjiang, China; 3 Key Laboratory of Modern Agricultural Equipment and Technology, Ministry of Education, Jiangsu University, Zhenjiang, China

**Keywords:** agricultural robot, crop load monitoring, deep learning, orchard management, orchard yield estimation, precision agriculture

## Abstract

Accurate yield estimation and crop load monitoring are essential for precision orchard management, supporting targeted fertilization, pruning, thinning, harvest planning, and marketing decisions. However, reliable *in-situ* monitoring remains challenging because commercial orchards are characterized by severe canopy occlusion, fruit overlap, heterogeneous tree architecture, variable illumination, and complex backgrounds. This review synthesizes advances in multi-modal sensing and deep learning for orchard yield estimation, breaking down the paradigm into intermediate fruit-counting or crop-load monitoring steps and supplementary spectral quality-assessment dimensions. First, yield-related indicators are summarized, including direct phenotypic traits such as fruit number, size, volume, and spatial distribution, as well as indirect structural and physiological proxies such as canopy volume, vegetation indices, flowering intensity, and spectral maturity attributes. Second, representative sensing devices and carrying platforms are reviewed, including red-green-blue (RGB) cameras, red-green-blue-depth (RGB-D) sensors, light detection and ranging (LiDAR), hyperspectral and multispectral systems, unmanned ground vehicles (UGVs), and unmanned aerial vehicles (UAVs). Third, the evolution of estimation methods is discussed, from traditional image processing and machine learning to object detection, instance segmentation, multi-object tracking, point-cloud analysis, remote-sensing regression, and multi-modal fusion. The review shows that no single sensor or algorithm can satisfy all orchard monitoring requirements. Ground-based vision and depth sensing are more suitable for fine-scale fruit counting and sizing, whereas UAV and spectral sensing provide advantages for regional yield mapping and quality-enhanced assessment. Future research should emphasize occlusion-aware perception, robust cross-environment generalization, lightweight edge deployment, standardized benchmarks, and integrated quantity–quality monitoring frameworks for actionable crop load management.

## 1 Introduction

The fruit tree industry constitutes a vital component of modern agriculture (Zhao et al., 2023a; Taha et al., 2025; Zhang et al., 2025a), wherein accurate yield information is essential for optimizing orchard productivity, improving resource use efficiency, and elevating economic returns (Ou et al., 2022; Guo et al., 2025a). In the paradigm of smart orchard management, precision yield and crop load estimation provide the indispensable data scaffolding for key localized interventions (Zhu et al., 2018; Lakhiar et al., 2020; Ge et al., 2025), including variable-rate fertilization, targeted pruning, precision flower and fruit thinning, and the logistical organization of automated harvesting (Ma et al., 2024b; Wang et al., 2025a, Wang et al., 2025b). Consequently, reliable yield estimation transcends mere production statistics; it functions as the foundational catalyst propelling the digital and intelligent transformation of fruit production systems (Dorj et al., 2017; Wang et al., 2025d).

Historically, conventional orchard yield estimation has predominantly relied upon manual visual assessment and localized statistical sampling (Wu et al., 2024; Johnson et al., 2025). These methodologies are intrinsically labor-intensive, time-consuming, and fundamentally fail to capture the profound spatial heterogeneity of large-scale commercial orchards (Koirala et al., 2019; Maheswari et al., 2021). To address these agronomic bottlenecks, recent advancements in multi-modal sensor technologies, machine vision, deep learning, and UAV remote sensing have catalyzed a profound paradigm shift (Wei et al., 2023). The technological trajectory has systematically evolved toward automated perception, predominantly diverging into two major pathways (Bargoti and Underwood, 2017): direct estimation (e.g., utilizing computer vision for fine-grained fruit detection and counting) and indirect estimation (e.g., leveraging canopy structural metrics, spectral responses, and machine learning models) (Zhou et al., 2020a; He et al., 2022; Cui et al., 2023). Longchamps et al. (2022) further expanded the research scope to cover both perennial and annual horticultural crops, systematically comparing deterministic and empirical yield sensing principles and highlighting the persistent gap between academic technological breakthroughs and large-scale commercial deployment of horticultural yield monitoring systems.

Nevertheless, executing high-precision yield estimation within complex orchards remains a formidable engineering challenge (Rashid et al., 2021). Authentic open-field environments are inherently unstructured, presenting severe perceptual hurdles such as dense branch and foliage occlusion, pronounced fruit overlapping, dynamic illumination variability, and cluttered homologous backgrounds (Linker, 2017; Wu et al., 2025a). These exogenous interferences frequently precipitate severe false negatives (missed detections), duplicate counting across video frames, unstable model inference, and constrained cross-scenario adaptability, thereby severely limiting the robust, large-scale deployment of autonomous perception platforms (Syed et al., 2025).

Although substantial progress has been achieved in the isolated domains of novel sensing devices and advanced perception algorithms, the existing literature remains highly fragmented (Häni et al., 2020; Wang et al., 2022b). Most previous reviews have focused on a single sensing modality, a specific algorithmic category, or a limited application scenario, with less attention paid to the systematic linkage among yield indicators, sensing platforms, algorithmic pathways, and field deployment requirements (Trentin et al., 2024). Compared with these studies, this review differs by linking yield-related indicators, sensing devices, carrying platforms, algorithmic methods, and deployment constraints within a unified task-oriented framework. In addition, this review emphasizes the practical suitability and limitations of different technologies under complex orchard conditions, such as occlusion, illumination variation, platform cost, and real-time requirements. Accordingly, this review is guided by the following research questions: (1) Which biological indicators are most relevant for orchard yield monitoring at fruit, tree, row, and orchard scales? (2) How do different sensing devices and carrying platforms match specific monitoring tasks under complex orchard conditions? (3) Which algorithmic methods are most suitable for fruit detection, counting, tracking, yield regression, and multi-modal fusion? (4) What technical, economic, and deployment constraints must be addressed before intelligent orchard yield monitoring can be widely adopted in real production systems?

The remainder of this review is organized as follows: Section 2 details the systematic literature search strategy and selection methodology. Section 3 introduces the biological monitoring indicators and standardized evaluation metrics related to orchard yield estimation. Section 4 comprehensively summarizes representative sensing devices and carrying platforms. Section 5 reviews the evolutionary trajectory of estimation models and methods, alongside a cross-method applicability analysis. Finally, Section 6 discusses current engineering challenges and future research prospects.

## 2 Literature search and selection methodology

To ensure a comprehensive and systematic synthesis of contemporary advancements, a rigorous literature retrieval protocol was executed across primary academic databases, predominantly comprising Web of Science (WoS), Scopus, and Google Scholar. Aligning with the explosive proliferation of deep learning and multi-modal sensing technologies in agricultural engineering, the temporal scope of the search was delineated from January 2016 to March 2026. The retrieval strategy employed a Boolean combination of definitive keywords encompassing three core dimensions: target tasks (“orchard yield estimation”, “crop load estimation”, “fruit detection”, “*in-situ* quality-enhanced yield”), perception hardware (“UAV”, “orchard robot”, “LiDAR”, “hyperspectral”), and algorithmic paradigms (“multi-object tracking”, “deep learning”, “instance segmentation”).

To maintain rigorous topical relevance, the initially retrieved records were subjected to stringent inclusion and exclusion criteria. The inclusion criteria mandated that studies must: (1) be explicitly deployed within authentic open-field orchard scenarios (in-situ); (2) directly address methodologies for absolute fruit quantification, crop load estimation, or quality-enhanced yield prediction (e.g., in-orchard spectral maturity monitoring); (3) propose or evaluate specific perception sensors, algorithmic architectures, or acquisition platforms; and (4) be peer-reviewed articles published primarily in English.

**Figure 1:**
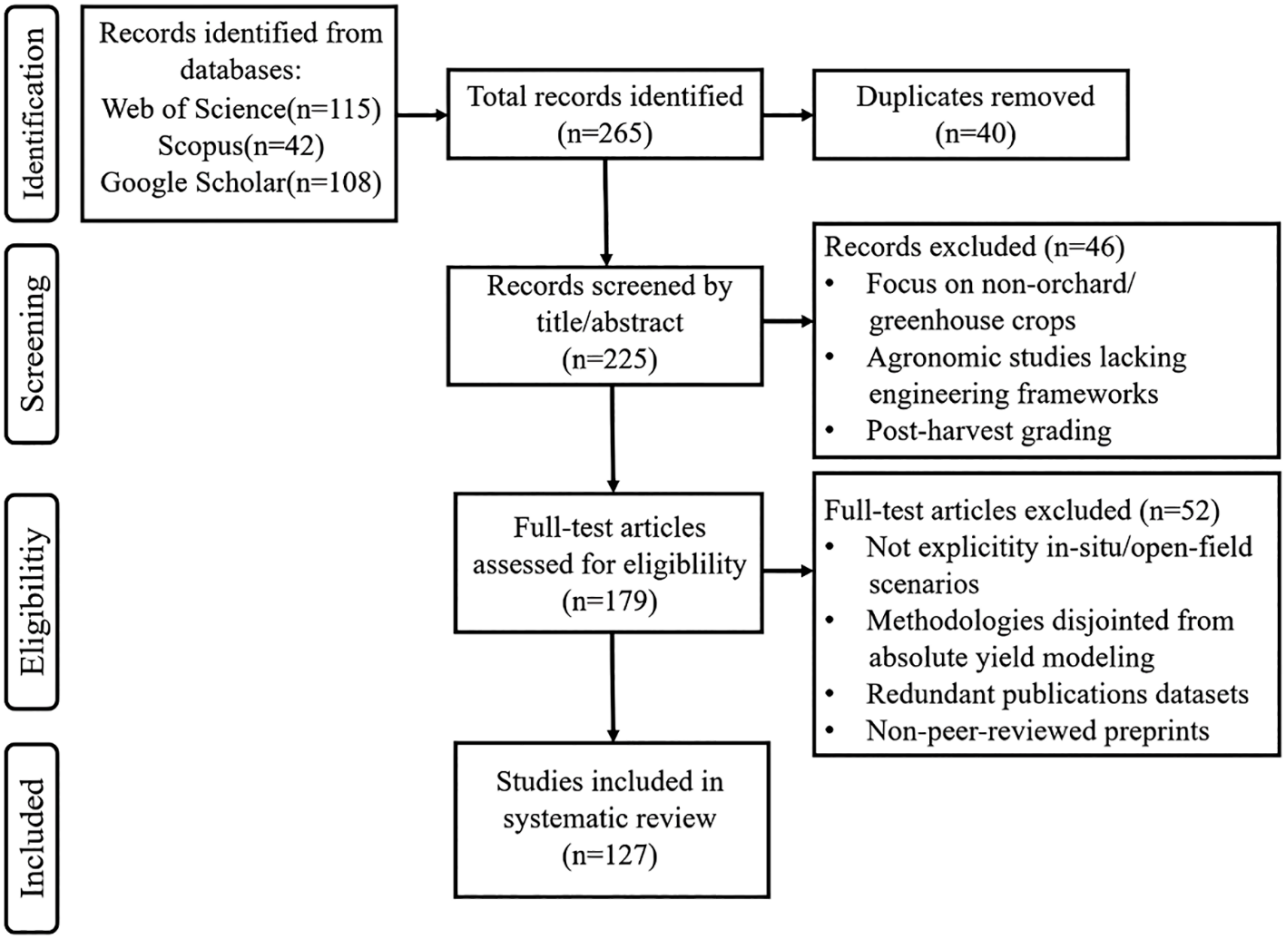
PRISMA flow diagram detailing the systematic literature retrieval and screening pipeline for studies on *in-situ* orchard yield estimation and crop load monitoring (Jan 2016 – Mar 2026).

Conversely, the exclusion criteria systematically filtered out: (1) research focused on greenhouse-cultivated vegetables or structured row crops; (2) post-harvest packing-house grading or static conveyor-belt evaluations that are entirely disjointed from pre-harvest yield modeling; (3) purely agronomic or physiological studies lacking engineering perception frameworks; and (4) redundant publications, duplicate datasets, or non-peer-reviewed preprints. The comprehensive step-by-step retrieval and screening pipeline is visually delineated in **Figure 1**.

## 3 Yield-related indicators and evaluation metrics

Precision yield estimation in orchards depends not only on sensing devices and detection algorithms but also on a clear understanding of yield-related biological indicators and a scientifically grounded evaluation framework (Gené-Mola et al., 2020). He et al. (2022) systematically summarized orchard fruit yield prediction from both direct fruit-level estimation and indirect proxy-based estimation perspectives, providing an important methodological basis for distinguishing counting-based and regression-based approaches. Building on this classification, the present review further links direct and indirect yield-related indicators with sensing devices, algorithmic pathways, and engineering deployment metrics. Instead, it involves the perception of direct yield-related traits, the integration of indirect proxy indicators, and the evaluation of model performance from detection accuracy to deployment feasibility (Guo et al., 2025b). Therefore, establishing a structured indicator system is essential for linking monitoring objectives with sensing modalities, algorithmic methods, and practical application scenarios (Palacios et al., 2023).

### 3.1 Direct yield-related indicators

**Figure 2:**
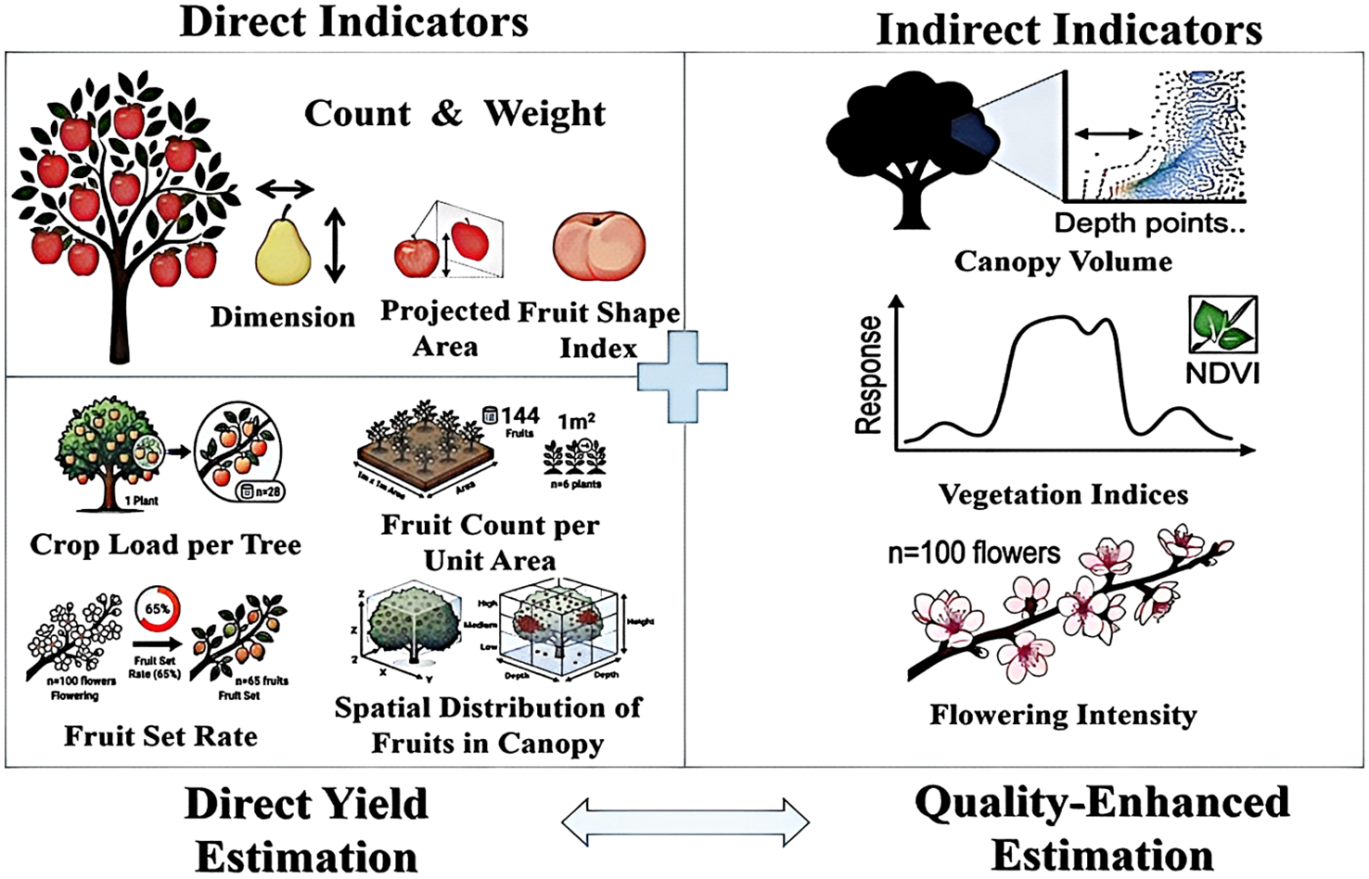
Direct and indirect monitoring pathways for orchard yield estimation and quality-enhanced assessment.

Direct yield-related indicators refer to variables that are directly associated with fruit quantity and harvestable yield (Tahir et al., 2018). These indicators mainly include fruit number, fruit size, fruit volume, and fruit spatial distribution within the canopy (Vasconez et al., 2020). In **Figure 2**, fruit number is the most intuitive and widely used variable in orchard yield estimation, as it provides the basis for crop load assessment at the tree, row, or orchard scale. However, fruit counting alone is often insufficient for accurate yield estimation, because overall yield is jointly determined by both the number of fruits and the mass of individual fruits.

Fruit size indicators, such as fruit diameter, fruit height, projected area, and shape index (Tran et al., 2023), provide important information for estimating single-fruit mass. Similarly, fruit volume serves as a more direct geometric descriptor for mass inversion and is particularly valuable when yield estimation requires moving beyond counting toward quantitative biomass prediction (Tu et al., 2020). In machine vision-based systems, the combination of fruit detection, dimension estimation, and volumetric inversion has gradually become a major technical pathway for improving estimation accuracy (Lin et al., 2021).

In addition to quantity and size, fruit spatial distribution within the canopy is also an important direct indicator (Mirhaji et al., 2021). The spatial arrangement of fruits affects visibility, sampling representativeness, and the robustness of detection and counting algorithms, especially in complex orchard environments with severe occlusion and uneven canopy structures (Jarron et al., 2020). Moreover, fruit distribution is closely related to tree architecture, pruning strategies, cultivar characteristics, and local microenvironmental conditions (Fernandes Jr and Yen, 2021; Zheng et al., 2022; He and Xiao, 2024). For this reason, recent studies have increasingly moved from simple fruit counting toward joint characterization of fruit number, fruit size, fruit volume, and spatial distribution, with the aim of better capturing the actual mechanism of yield formation. It should be noted that these direct indicators do not always contribute equally to marketable yield; for instance, fruit size and quality may have a larger impact than fruit number in determining the final marketable harvest.

### 3.2 Indirect yield-related indicators

In addition to direct fruit-level traits, orchard yield can also be inferred from indirect indicators that reflect canopy structure, physiological status, or reproductive potential (Dechant et al., 2020; Lee et al., 2021). These indicators mainly include canopy volume, vegetation indices, spectral maturity proxies, and flowering intensity. Although they do not directly measure harvestable fruit yield, they provide complementary information for yield prediction, particularly at larger spatial scales or in scenarios where direct fruit observation is difficult (Zhou et al., 2021; Agustí et al., 2022; Zeng et al., 2022).

Canopy volume is one of the most representative structural indicators. It reflects tree vigor, canopy architecture, and light interception capacity, and is often associated with fruit-bearing potential and biomass accumulation (Ferreira et al., 2026). In orchard yield estimation, canopy volume is commonly derived from RGB-D data, LiDAR point clouds, or UAV-based 3D reconstruction (Fayad et al., 2021), and is frequently used as an explanatory variable in tree-level or plot-level yield models.

Vegetation indices, such as the normalized difference vegetation index (NDVI) and related multispectral indicators, are widely used to describe canopy greenness, vigor, and photosynthetic activity (Gao et al., 2024). These indices are particularly useful in UAV-based or remote sensing-based yield estimation, where they serve as proxies for crop growth status and spatial variability across orchards. Similarly, flowering intensity is an important early-season indicator because flowering density is strongly related to potential fruit set and subsequent yield formation. Monitoring flower abundance can therefore provide an early basis for pre-harvest yield forecasting (Yan et al., 2022).

Another important category of indirect indicators is spectral maturity proxies. During fruit development and ripening, changes in peel pigments, moisture, soluble solids content, dry matter, and internal cellular structure alter the optical response of fruits across the visible, near-infrared, and short-wave infrared bands (Galal et al., 2022; Gómez-Lagos et al., 2023). As a result, spectral measurements can be used to infer maturity status and internal quality attributes. From the perspective of yield estimation, these indicators are particularly useful for quality-enhanced yield prediction and harvest timing assessment (Gao et al., 2020; Huang et al., 2024), because fruit maturity is often associated with single-fruit mass accumulation and marketable yield. Nevertheless, such physiological indicators are indirectly related to final yield and are better regarded as complementary predictors rather than substitutes for quantitative fruit detection.

### 3.3 Evaluation metrics

A standardized evaluation system is essential for comparing different orchard yield estimating methods and for linking algorithmic performance with practical deployment requirements. In this review, evaluation metrics are grouped into four categories: detection metrics, counting metrics, regression metrics, and deployment metrics (Xiong et al., 2020) (**Table 1**).

**Table 1:** Summary of evaluation metrics for orchard yield estimation models.

Dimension	Metric	Abbreviation	Core Formula	Applicable Task	Function
Detection	Precision and Recall	P/R	$\text{P}=\frac{\text{TP}}{\text{TP}+\text{FP}}$ $\text{R}=\frac{\text{TP}}{\text{TP}+\text{FN}}$	Fruit detection/instance segmentation	Measures the mitigation of false negatives and false positives.
	F1-score	F1	$\text{F}1=\frac{2\text{PR}}{\text{P}+\text{R}}$	Fruit detection/instance segmentation	Represents the harmonic mean of precision and recall.
	Average Precision	AP	$\text{AP}=\int_{0}^{1}\text{P}(\text{R})\text{dR}$	Fruit detection/instance segmentation	Evaluates detection efficacy for a specific single category.
	mean Average Precision	mAP	$\text{mAP}=\frac{1}{\text{C}}\sum_{\text{j}=1}^{\text{c}}{\text{AP}}_{\text{j}}$	Fruit detection/instance segmentation	Quantifies overall recognition capability across all categories.
Counting	Counting accuracy	CA	$\text{CA}=1-\frac{\left|{\text{N}}_{\text{p}}-{\text{N}}_{\text{g}}\right|}{{\text{N}}_{\text{g}}}$	Fruit counting/video-based counting	Reflects the agreement between predicted and ground-truth fruit counts
	Counting error rate	CER	$\text{CER}=\frac{\left|{\text{N}}_{\text{p}}-{\text{N}}_{\text{g}}\right|}{{\text{N}}_{\text{g}}}$	Fruit counting/video-based counting	Quantifies the relative counting error between predicted and ground-truth counts
	Multiple Object Tracking Accuracy	MOTA	$\text{MOTA}=1-\frac{\sum_{\text{t}}({\text{FN}}_{\text{t}}+{\text{FP}}_{\text{t}}+{\text{IDS}}_{\text{t}})}{\sum_{\text{t}}{\text{GT}}_{\text{i}}}$	Video-based counting/MOT	Measures overall tracking and counting accuracy by considering missed detections
	Multiple Object Tracking Precision	MOTP	$\text{MOTP}=\frac{\sum_{\text{i},\text{t}}{\text{d}}_{\text{i},\text{t}}}{\sum_{\text{i}}{\text{c}}_{\text{t}}}$	Video-based counting/MOT	Measures localization precision of tracked objects
Regression	Coefficient of determination	R^2^	${\text{R}}^{2}=1-\frac{\sum_{\text{i}=1}^{\text{n}}{\left({\text{y}}_{\text{i}}-\hat{{\text{y}}_{\text{i}}}\right)}^{2}}{\sum_{\text{i}=1}^{\text{n}}{\left({\text{y}}_{\text{i}}-\bar{\text{y}}\right)}^{2}}$	Yield regression/mass estimation	Assesses goodness of fit and explanatory variance for yield.
	Root mean square error	RMSE	$\text{RMSE}=\sqrt{\frac{1}{\text{n}}\sum_{\text{i}=1}^{\text{n}}{\left({\text{y}}_{\text{i}}-\hat{{\text{y}}_{\text{i}}}\right)}^{2}}$	Yield regression/mass estimation	Quantifies the absolute error magnitude of predictions.
	Mean absolute error	MAE	$\text{RAE}=\frac{1}{\text{n}}\sum_{\text{i}=1}^{\text{n}}\left|{\text{y}}_{\text{i}}-\hat{{\text{y}}_{\text{i}}}\right|$	Yield regression/mass estimation	Reflects the linear average of prediction deviations.
Deployment	Parameters	Params	-	Edge deployment/model comparison	Dictates the model’s memory footprint.
	Floating-point operations	FLOPs	-	Edge deployment/model comparison	Indicates theoretical computational complexity.
	Frames per second	FPS	-	Real-time detection/onboard inference	Evaluates real-time processing capability for mobile deployment.

-, not specified.

Detection metrics are mainly used for fruit detection and instance segmentation tasks. Common indicators include Precision, Recall, F1-score, Average Precision, and mean Average Precision (Zhou et al., 2023), which are used to evaluate the correctness, completeness, and overall recognition capability of detection models (Gao et al., 2025b). These metrics are particularly important in complex orchards, where severe occlusion, fruit overlap, and background clutter can easily lead to missed detections and false positives (Jin et al., 2021).

Counting metrics are especially relevant for video-based fruit counting and multi-object tracking tasks (Li et al., 2021). In addition to counting accuracy and counting error rate, commonly used tracking indicators include Multiple Object Tracking Accuracy, Identification F1 score, and Multiple Object Tracking Precision (Yu et al., 2024). These metrics reflect different aspects of counting performance, including duplicate counting, missed counting, identity consistency, and localization quality during continuous observation. Since orchard video counting often relies on tracking fruits across frames to avoid repeated counts, these metrics are essential for evaluating the effectiveness of tracking-based methods.

Regression metrics are mainly used for yield estimation, fruit mass prediction, and yield inversion tasks (Rosado et al., 2022; Johnson et al., 2025). Representative indicators include the coefficient of determination, root mean square error, mean absolute error, and mean absolute percentage error, which reflect explanatory ability, average prediction deviation, and relative estimation error. These metrics are suitable for evaluating models that directly predict fruit yield, crop load, or related continuous variables.

Deployment metrics are used to assess the engineering feasibility of sensing and estimation systems, especially for mobile robots, UAVs, and edge devices (Fleming et al., 2024; Liu et al., 2024b). Typical indicators include the number of model parameters, floating-point operations, inference speed measured by frames per second, and energy consumption. These metrics are critical for judging whether a method can satisfy the constraints of real-time operation, onboard computation, and large-scale field deployment.

Orchard yield monitoring can be understood as an integrated pipeline. Monitoring objectives first determine the selection of yield-related indicators; these indicators then guide the choice of sensing devices and carrying platforms; the acquired data further determine suitable algorithmic methods; and practical deployment constraints finally influence system-level applicability. This indicator–sensor–platform–algorithm–application logic provides the conceptual basis for the following sections.

In summary, fruit morphological features establish the core phenotypic foundation for direct yield estimation, spectral responses furnish the physiological basis for quality-enhanced yield estimation, and multi-dimensional evaluation metrics constitute a unified standardized framework for benchmarking the performance of diverse monitoring schemes. Only when predicated upon these fundamental mechanisms and evaluation indicators can subsequent data acquisition and sensing technologies construct an effective monitoring system tailored for complex orchards.

## 4 Equipment and carrying platforms

**Figure 3:**
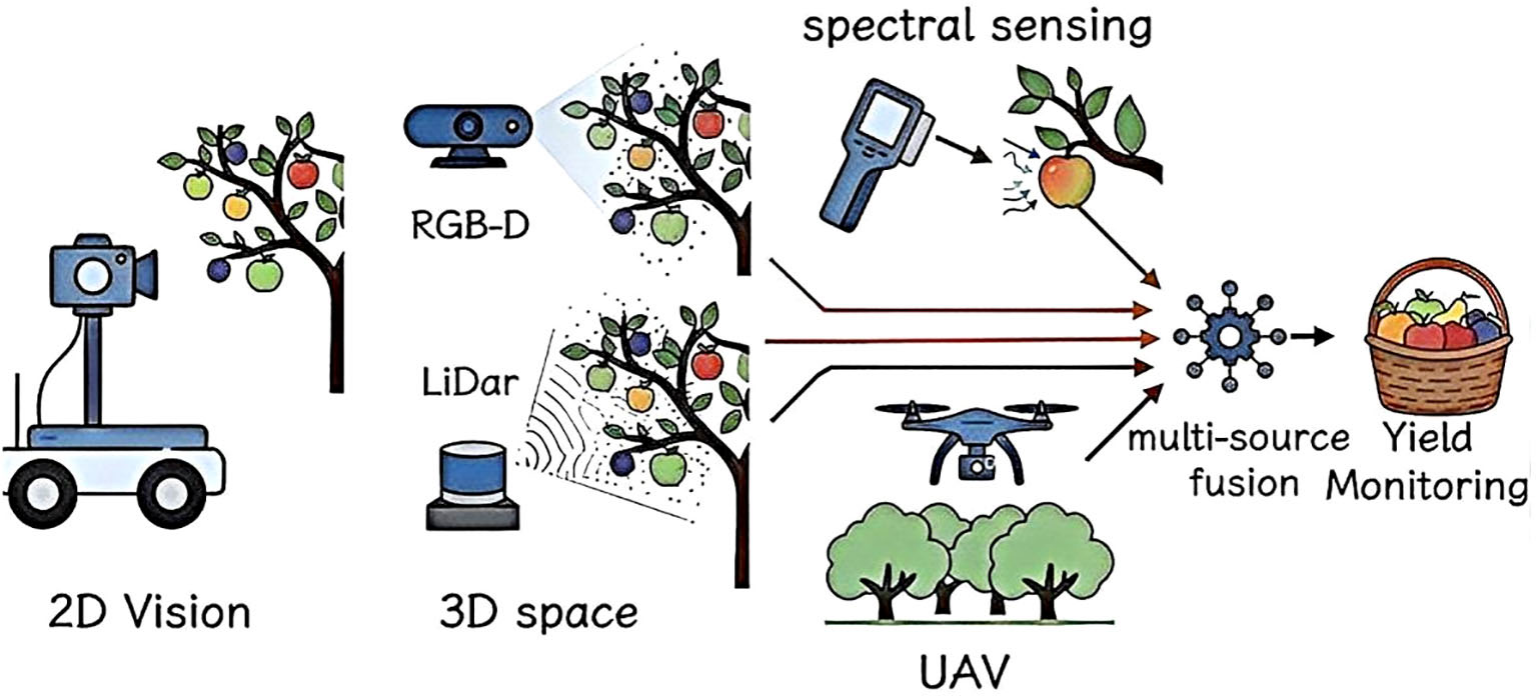
Representative sensing devices and carrying platforms for orchard yield monitoring.

In **Figure 3**, orchard environments are inherently characterized by typical unstructured features, including dense fruit overlapping, severe branch and foliage occlusion, and drastic natural illumination variations (Apolo-Apolo et al., 2020; Lei et al., 2023). However, single-sensor modalities inevitably suffer from perceptual blind spots, rendering them highly susceptible to missed detections and inadequate to satisfy the stringent requirements for all-weather, high-precision yield estimation (Osman et al., 2021). Rong et al. (2026) recently provided an updated overview of multi-source information perception sensors for precision orchard yield estimation, quantitatively comparing the performance of different sensor combinations and emphasizing that multi-modal fusion is the most promising direction to address the inherent limitations of single-sensor systems in complex orchard environments. Therefore, sensor selection in orchard yield monitoring should be scenario-dependent rather than technology-driven. RGB cameras are more suitable for low-cost fruit detection under moderate occlusion, RGB-D and LiDAR sensors are preferable when spatial localization and canopy structure are required, spectral sensors are more appropriate for maturity or quality-related assessment, and UAV platforms are better suited for large-scale canopy-level yield mapping rather than fine-scale fruit counting.

### 4.1 2D vision-based yield estimation

#### 4.1.1 Static RGB imaging devices

**Figure 4:**
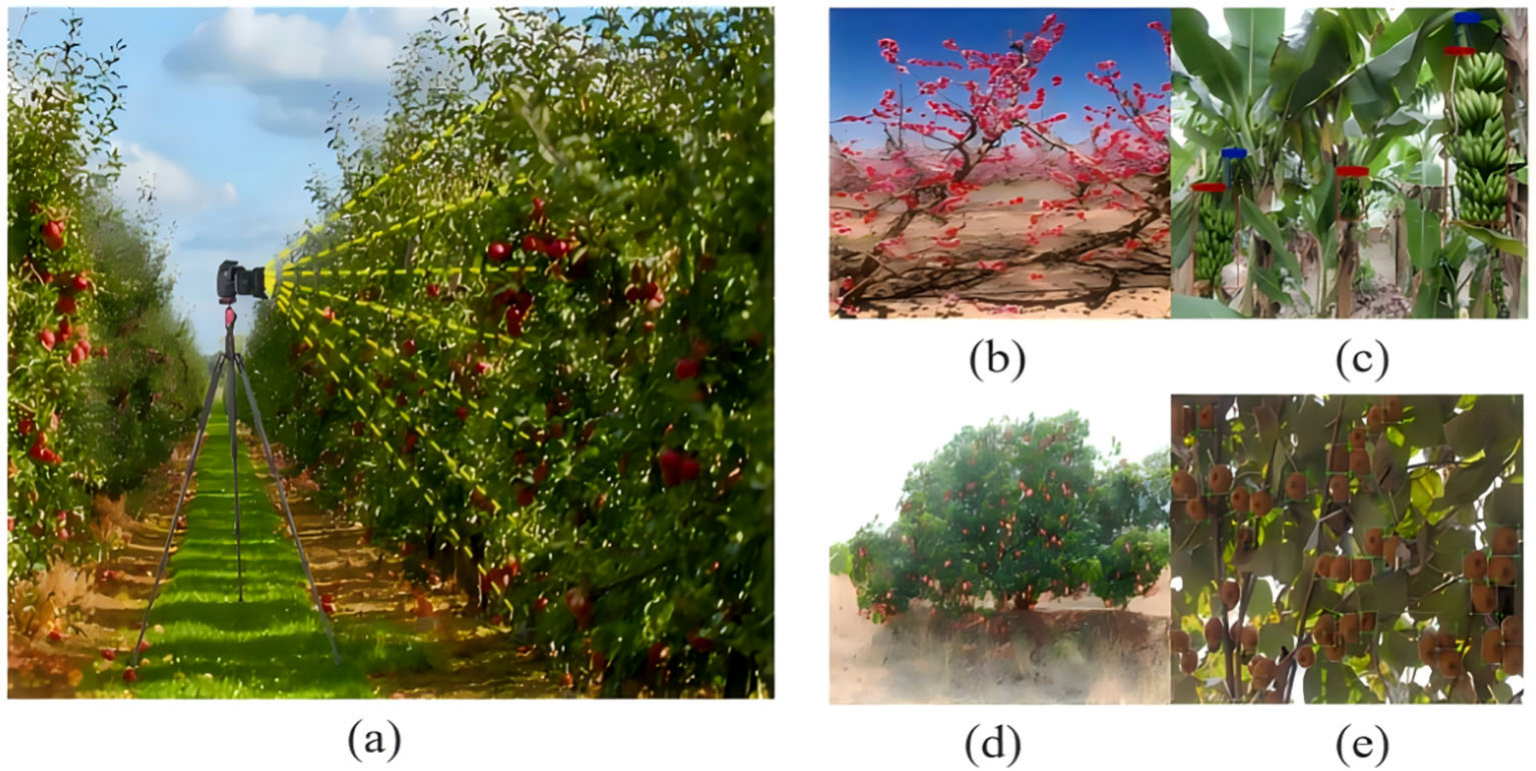
**(a)** Static collection of RGB images (Tan et al., 2025); **(b–e)** RGB yield estimation interfaces for different fruit trees (Mekhalfi et al., 2020; Fu et al., 2022; Estrada et al., 2024; Zhang et al., 2025a).

**Figure 5:**
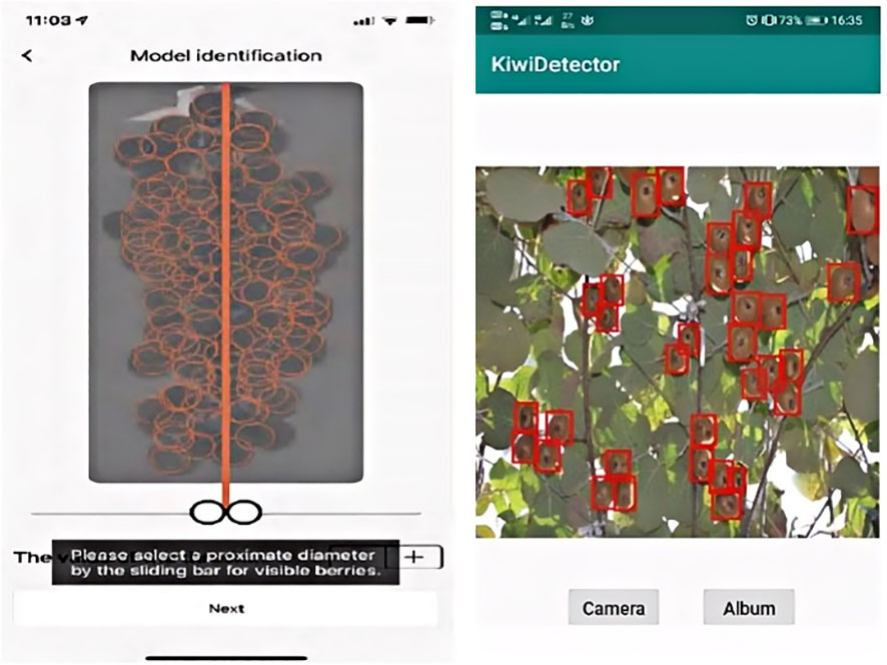
Portable smartphone RGB image yield estimation application. **(a)** Grape detection APP interface (Liu et al., 2020); **(b)** Kiwifruit detection APP interface (Zhou et al., 2020b).

RGB cameras represent the most universally adopted and cost-effective foundational vision apparatuses in orchard yield estimation (Peng et al., 2021; Jiang et al., 2025). Owing to their hardware maturity, deployment flexibility, and high spatial resolution, conventional industrial cameras, consumer-grade digital cameras, and smartphone lenses can all serve as near-ground yield estimation devices (as illustrated in **Figures 4**, **5**). Extensive literature indicates that fruit detection systems predicated on static RGB images have been extensively applied in yield estimation studies for diverse crops, notably apples, citrus, and mangoes. From a task-oriented perspective, static RGB devices are optimally suited for low-cost absolute fruit counting and single-tree crop load estimation via edge computing or smartphones. While they exhibit exceptionally high deployment readiness and operational flexibility, their reliance on pure 2D phenotypic information fundamentally restricts their efficacy in complex canopies (Divyanth et al., 2023). Specifically, the intrinsic absence of depth data renders them highly susceptible to severe branch/foliage occlusion, frequently precipitating redundant counting and false positives. In addition, static RGB acquisition is prone to sampling bias and view-angle dependence, because fruit visibility varies substantially with camera position, canopy side, and imaging distance. Image overlap and occluded or hidden fruits further limit the reliability of fruit counting, particularly in dense canopies where fruits located inside the canopy are difficult to estimate from a single 2D view (Zhao et al., 2023b).

#### 4.1.2 Ground-based carrying platforms (UGVs)

**Figure 6:**
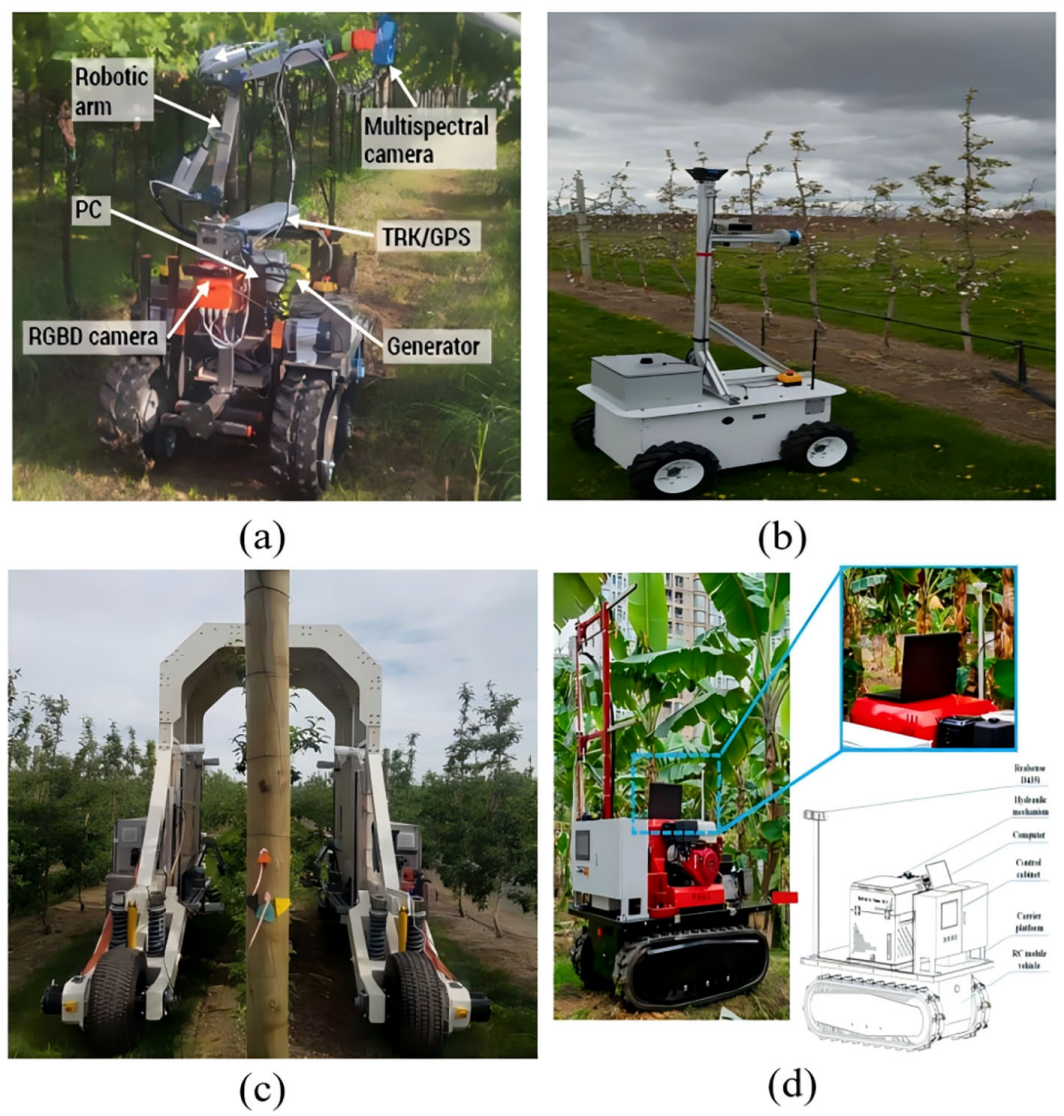
Representative ground-based platforms and unmanned ground vehicles (UGVs) utilized for precision yield estimation. **(a)** autonomous platform for yield monitoring in orchard (Visentin et al., 2024); **(b)** mobile platform for fruit flower cluster detection (Lee et al., 2022); **(c)** robotic platform for apple scanning (Qureshi et al., 2023); **(d)** carrying platform for banana yield estimation (Zhou et al., 2025).

Ground-based platforms, particularly unmanned ground vehicles (UGVs), constitute indispensable carriers for proximal-range data acquisition (Ahmed et al., 2022; Yin et al., 2025). In contrast to static point-sampling methodologies, UGVs facilitate continuous operation along tree rows, typically integrating industrial cameras, LiDAR, and GNSS units shown in **Figure 6**. Operationally, these platforms are highly suited for continuous tree-row scanning, dynamic fruit tracking, and cross-frame redundant counting mitigation, providing critical decision support for early-stage flower cluster detection and yield forecasting (Lee et al., 2022). Nevertheless, continuous operation entails substantial data redundancy and intensified real-time processing requirements (Zhang et al., 2025c). Ultimately, the effective deployment of these systems remains contingent upon the synergistic optimization of platform mechanical stability and robust backend tracking algorithms to mitigate motion blur and drastic illumination variation.

### 4.2 3D spatial-based yield estimation

Owing to the intrinsic absence of depth information in 2D imagery, severe branch and foliage occlusion alongside fruit overlapping constitute the fundamental drivers of missed detections (false negatives) and false positives 3D spatial perception technologies effectively circumvent these limitations by introducing a third dimension to the sensing manifold.

#### 4.2.1 RGB-D sensors

In **Table 2**, RGB-D sensors facilitate the acquisition of augmented spatial data by simultaneously outputting RGB imagery and corresponding depth maps (Gao et al., 2025a; Ma et al., 2025; Lee et al., 2026). Task-wise, this extra dimension provides essential support for precise fruit spatial localization, size/volume estimation, and close-range 3D perception to effectively mitigate occlusion bottlenecks. However, the performance of these devices (particularly structured light and Time-of-Flight sensors) significantly degrades under strong outdoor sunlight and high reflectivity (Tsoulias et al., 2022; Khan et al., 2025). Consequently, their effective deployment is strictly restricted to near-field ranges (typically < 5 meters) or environments with controlled illumination (Farhan et al., 2024).

**Table 2:** Technical specifications of representative RGB-D sensors.

Manufacturer	Stereolabs	Microsoft	Intel	Orbbec
Model	ZED 2i	Kinect V2	Real Sense D435i	Gemini 2L
Appearance	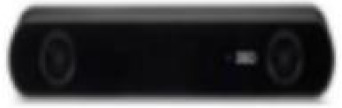	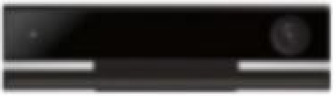	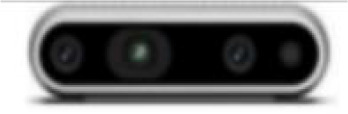	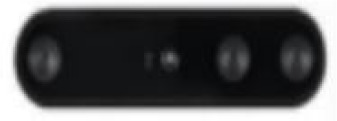
Working Principle	Stereo Vision	Time-of-Flight	Structured Light	Structured Light
Depth Range (m)	0.3m-3m	0.4m-4m	0.2m-2m	0.25m-7m
Field of View (FoV)	110°×70°×120°	70°×60°×100°	87°×58°×95°	91°×66°×101°

#### 4.2.2 LiDAR scanning and point cloud acquisition platforms

**Figure 7:**
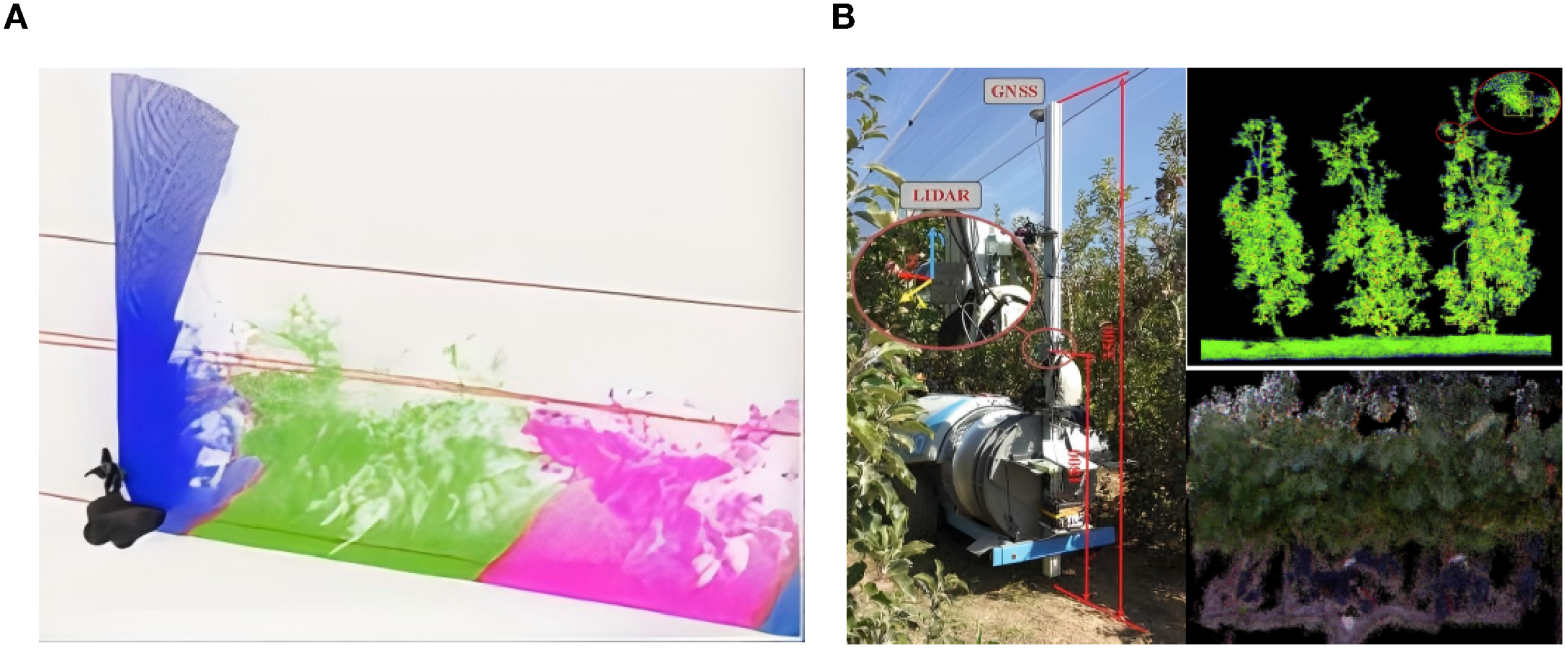
Representative 3D spatial information acquisition platforms: **(a)** LiDAR scanning platform (Underwood et al., 2016); **(b)** 3D point cloud acquisition platform (Gené-Mola et al., 2019a).

LiDAR scanning platforms (such as Mobile Terrestrial Laser Scanning) serve as critical milestones in the paradigm shift towards spatial structural perception (Murcia et al., 2021). By utilizing active laser emission, they directly capture the high-fidelity 3D spatial coordinates and geometric morphology of target objects, effectively transcending the “surface-only” constraints of 2D imagery shown in **Figure 7** (Zhenyu et al., 2025). Therefore, LiDAR is exceptionally ideal for canopy structural analysis, 3D spatial distribution mapping, and robust volume extraction. Despite their illumination-invariant nature, widespread autonomous deployment is currently hampered by prohibitive hardware costs, computationally complex point cloud processing, and the inherently sparse fruit-level resolution compared to RGB pixel density (Zhang et al., 2025b).

### 4.3 Spectral sensing devices

In contrast to RGB and 3D visual apparatuses, proximal spectral sensing devices (e.g., visible/near-infrared (Vis/NIR) spectrometers and hyperspectral imaging (HSI) systems) possess the unique capability to extract internal physiochemical parameters—including soluble solids content (SSC), dry matter (DM), and firmness—via the reflectance characteristics of the fruit peel (Ram et al., 2024; Chaudhary et al., 2025). This physiological sensing capability drives a crucial paradigm shift in yield monitoring systems: transitioning from mere “quantitative counting” to “synergistic quantity-quality evaluation”. Strategically, portable NIR spectrometers are optimally deployed for *in-situ* maturity assessment, quality-enhanced yield estimation, and optimal harvest scheduling (Tian et al., 2022; Lin et al., 2026).

**Figure 8:**
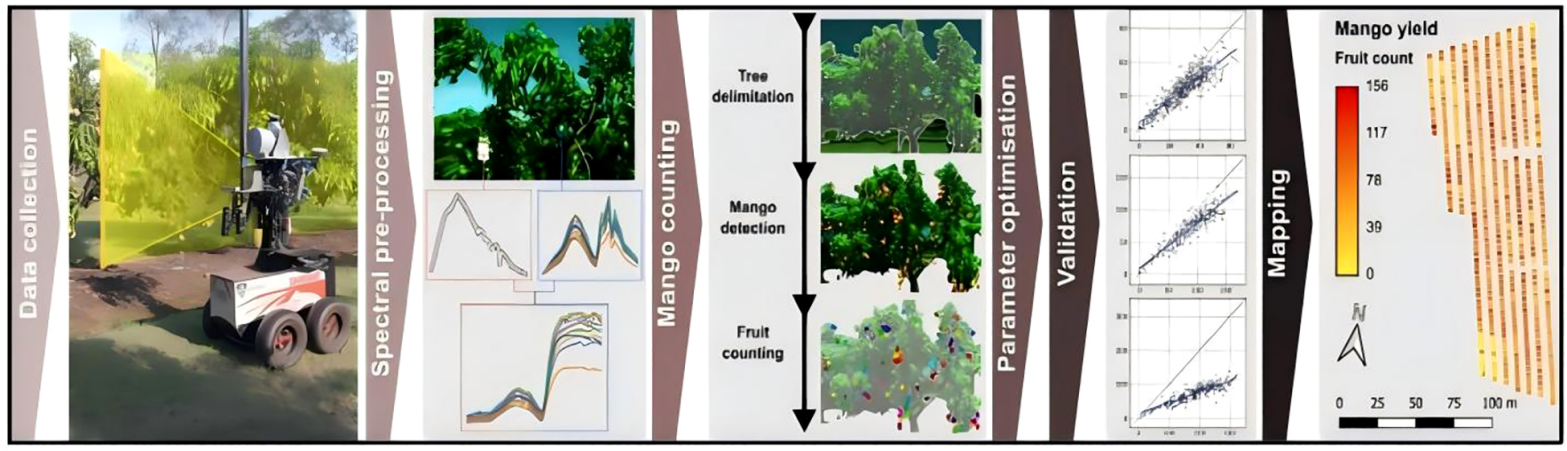
Yield estimation pipeline based on hyperspectral imaging (Gutiérrez et al., 2019).

Spectral sensing is rapidly expanding from single-fruit assessment towards orchard-scale multi-modal fusion. In **Figure 8**, ground-based hyperspectral imaging (HSI) integrated with UGVs has demonstrated the potential for continuous crop load estimation, achieving robust correlations (R^2^>0.75) while generating spatial yield maps (shown in **Figure 7**) (Gutiérrez et al., 2019). Furthermore, fusing multispectral vegetation indices (e.g., NDVI) with RGB color features significantly augments cross-frame object tracking and maturity classification (Pourdarbani et al., 2020). Such multi-modal frameworks synergize superficial phenotypic data with deep physiological dimensions, seamlessly mitigating redundant counting while optimizing harvest decisions (Pan and Liu, 2026).

Crucially, the engineering deployment of spectral devices within complex orchards confronts severe physical and computational constraints. Beyond the massive data dimensionality and cost, HSI remains fundamentally a 2D imaging modality. Restricted by narrow fields of view (FOV) and pushbroom (line-scan) imaging mechanisms, HSI struggles to facilitate multi-view 3D reconstruction or completely circumvent missed detections (false negatives) induced by severe canopy occlusion. Consequently, while highly viable for specialized multi-objective monitoring (e.g., maturity and disease), a discernible gap persists between current spectral technologies and the stringent requirements for low-cost, real-time absolute fruit counting (Li et al., 2018).

### 4.4 Low-altitude remote sensing platforms

**Figure 9:**
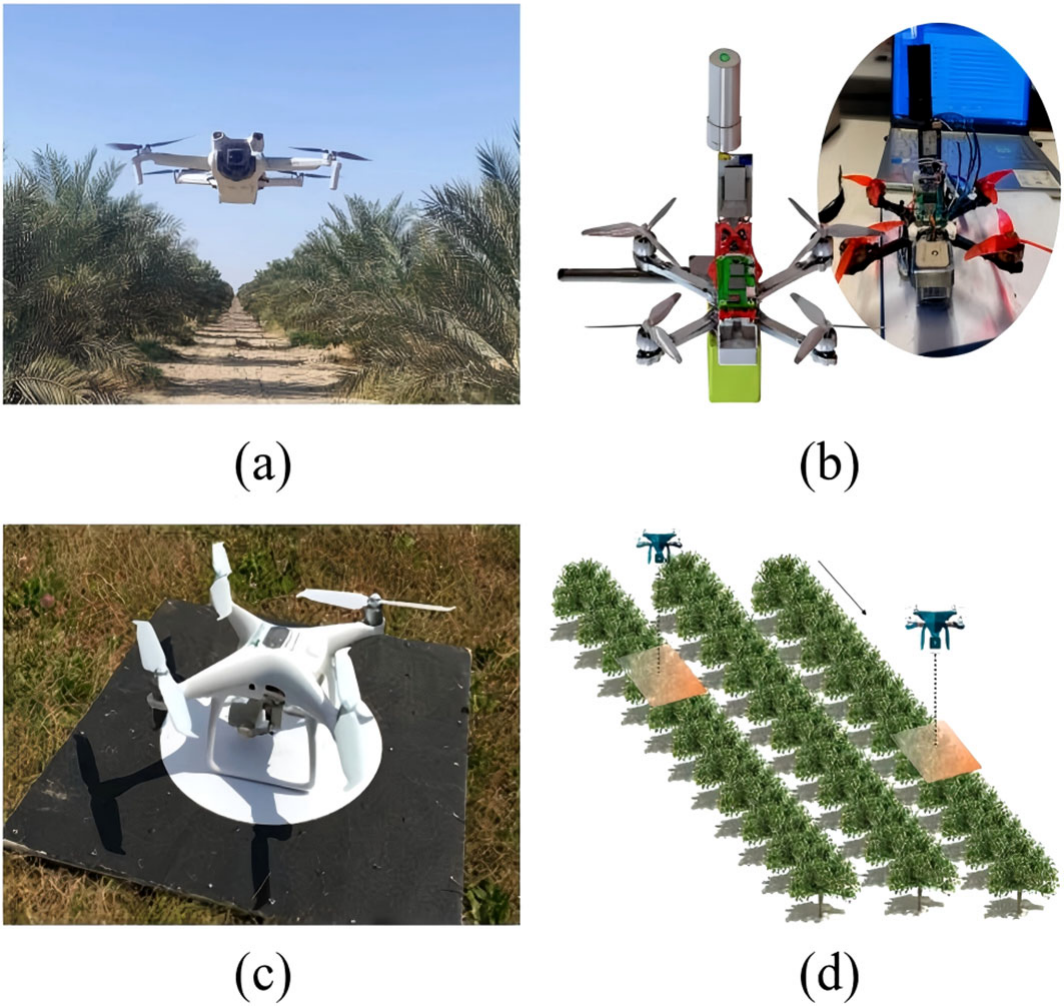
**(a–d)** Representative unmanned aerial vehicle (UAV) remote sensing platforms utilized for orchard estimation (Apolo-Apolo et al., 2020; Kodors et al., 2024; Al-Abbas et al., 2026).

In **Figure 9**, tailored to specific mission requirements, UAV platforms can be flexibly integrated with a diverse array of sensors—encompassing RGB cameras, multispectral imagers, thermal infrared sensors, and LiDAR—to systematically acquire canopy imagery, vegetation indices (VIs), thermal distributions, 3D structural topologies, and spatial heterogeneity metrics (Subeesh et al., 2024; Zhang et al., 2025d). Ozdarici-Ok and Ok (2023) reviewed remote sensing-based individual tree species identification methods in orchards, which provides critical foundational support for UAV-based yield estimation by enabling species-specific model fine-tuning and accurate single-tree canopy segmentation in mixed-variety orchard scenarios. In stark contrast to ground-based systems, UAV platforms are distinguished by their expansive field of view (FOV), superlative operational efficiency, and extensive single-flight spatial coverage (Afsar et al., 2025). These attributes render them exceptionally viable for high-throughput data acquisition within large-scale commercial orchards, topologically complex hilly/mountainous terrains, and regions where manual inspection incurs prohibitive financial costs. Furthermore, within orchard environments characterized by uniform canopy architectures and distinctly delineated planting rows, UAVs can expeditiously execute whole-orchard monitoring (Ji et al., 2021; Li et al., 2023). This macro-level capability provides robust data scaffolding for canopy fractional cover analysis, 3D canopy volume extraction, vegetative vigor zoning, and regional yield estimation (Zhang et al., 2022).

Nevertheless, UAV platforms possess inherent and distinct limitations. Constrained predominantly by a top-down (nadir) viewing angle, fruits situated within the inner canopy are frequently occluded by dense foliage (Feng et al., 2024), rendering direct observation exceedingly difficult. Consequently, their efficacy in single-tree crop load quantification and inner-canopy fruit recognition remains fundamentally restricted (Li et al., 2025b). In addition, flight altitude directly affects the trade-off between spatial coverage and ground sampling distance: lower altitudes can provide higher spatial resolution for canopy- or fruit-level observation but reduce coverage efficiency, whereas higher altitudes improve orchard-scale coverage but may lose fine fruit-level details. For multispectral or hyperspectral UAV monitoring, radiometric calibration is also necessary to ensure the reliability of vegetation indices, maturity assessment, and cross-date comparison. Concurrently, UAV operations are highly susceptible to exogenous factors, including adverse weather conditions (Zhang et al., 2019; Jiang et al., 2026b), flight altitude restrictions (Ahmed et al., 2021), limited battery endurance, and stringent aviation safety regulations (Zhu et al., 2022). Furthermore, subsequent data processing pipelines—encompassing image stitching, orthorectification, and 3D reconstruction—are computationally intensive and procedurally complex. Ultimately, it is evident that while UAV platforms are exemplary tools for macroscopic perception and regional monitoring, they cannot autonomously supplant ground-based (proximal) platforms in the execution of fine-grained precision yield monitoring.

### 4.5 Task-oriented comparison of sensing devices

To systematically summarize the complementary nature of the aforementioned technologies, **Table 3** provides a comprehensive, task-oriented comparison of representative sensing devices, delineating their functional strengths, applicable scales, and deployment readiness for precision yield estimation.

**Table 3:** Task-oriented comparison of representative sensing devices for orchard yield estimation.

Sensor modality	RGB camera	RGB-D sensor	LiDAR	Hyper/ multi-spectral	UAV platform
Output Data Type	2D pixels (Color, texture)	RGB + Depth map	3D Point Cloud	Spectral signatures, VIs	Orthomosaics, Aerial point clouds
Typical Range	Proximal to Mid	Near-field (< 5m)	Mid to Long	Proximal to Aerial	Aerial (Macro)
Primary Strength	High resolution, low-cost, mature algorithms	Direct distance, enables 3D spatial separation	Illumination-invariant, robust structural penetration	Captures internal physiological traits (SSC, DM)	High-throughput, large coverage, no terrain constraints
Primary Limitation	Severe occlusion, no depth, redundant counting	Sensitive to strong outdoor sunlight/reflectivity	Sparse fruit-level resolution, complex processing	Massive data redundancy, narrow FOV	Inner-canopy occlusion, low single-fruit accuracy
Suitable Scale	Single-fruit/Single-tree	Single-fruit/Local canopy	Single-tree/Row level	Single-fruit/Regional	Whole-orchard/Regional
Optimal Yield Task	Low-cost counting, crop load estimation	Fruit localization, size/volume estimation	Canopy structural analysis, 3D distribution	Maturity assessment, quality-enhanced estimation	Regional yield mapping, spatio-temporal monitoring
Deployment Readiness	High (Edge devices, smartphones)	High (Requires illumination control)	Moderate (Restricted by hardware cost)	Moderate (Mainly *in-situ* sampling)	High (For macro-scale spatial mapping)

## 5 Models and methods

The trajectory of orchard yield estimating methodologies illustrates a continuous evolution of yield estimation technologies within complex orchards: transitioning from empirical rule-driven paradigms to data-driven approaches, and progressively advancing towards multi-source synergistic frameworks. Early investigations predominantly relied upon hand-crafted rules—leveraging color, texture, and morphological features—to execute fruit recognition and counting. Subsequently, researchers introduced traditional machine learning models, integrating hand-crafted features with classification and regression algorithms, thereby mitigating interference and enhancing yield estimation capabilities against complex backgrounds (Albaaji et al., 2025). In recent years, deep learning methodologies, notably convolutional neural networks (CNNs), object detection, and instance segmentation, have emerged as the prevailing mainstream. This paradigm is further evolving towards multi-modal fusion (Gené-Mola et al., 2019b; Sun et al., 2020; Guo et al., 2025b), integrating remote sensing, environmental, and temporal data to facilitate more comprehensive analyses.

### 5.1 Traditional yield estimation methods

Traditional orchard yield estimation mainly includes two technical paradigms: rule-based image processing and hand-crafted feature-based machine learning (Häni et al., 2018). Early studies primarily relied on color space transformation, threshold segmentation, and morphological operations to extract fruit regions under relatively simple conditions. Representative methods further incorporated shape priors and boundary detection strategies, such as circular fitting and Hough transform, to improve fruit localization and counting performance (Pichhika et al., 2025). To reduce environmental interference, subsequent studies introduced optimized feature extraction schemes in combination with statistical models, including improved thresholding methods, texture descriptors, and manually designed features such as GLCM, HOG, and Haar-like features, which were then coupled with classifiers such as SVM, Random Forest, AdaBoost, and K-means clustering for fruit detection, separation, and related estimation tasks (Zhang et al., 2021).

Although these methods achieved satisfactory results in controlled scenarios with simple backgrounds and clear fruit contours, they remained strongly dependent on empirical rules and manual feature design. Their performance is therefore highly sensitive to illumination changes, canopy occlusion, fruit overlap, and background similarity, which are common in real orchard environments (Anderson et al., 2021; Oikonomidis et al., 2023). As orchard monitoring tasks expanded from constrained single-image analysis to continuous field deployment on mobile platforms, the limited robustness and generalization ability of traditional methods became increasingly evident. These limitations ultimately drove the transition from manual feature engineering to automatic feature learning, laying the foundation for modern deep learning-based yield estimating frameworks.

### 5.2 Deep learning methods for yield estimation

End-to-end deep learning has emerged as the dominant paradigm for orchard yield estimation, enabling robust fruit detection, counting, and yield prediction under complex field conditions (Wang et al., 2022a; Zheng et al., 2026). Unlike traditional approaches, deep networks autonomously learn hierarchical, discriminative features across multiple scales, reducing reliance on hand-crafted features and improving robustness to dense canopy occlusion, fruit overlap, complex backgrounds, and variable illumination. This automated representation is particularly important in real orchards, where fruit morphology and canopy structure vary substantially and empirical rules often fail to generalize.

Deep learning-based methods can be broadly categorized into three trajectories. Object detection-based approaches efficiently localize and count individual fruits (Ji et al., 2022; Lei et al., 2025), making them suitable for rapid tree- or row-level yield estimation (Raza et al., 2024). Semantic and instance segmentation methods provide pixel-level delineation, with semantic segmentation effective for canopy coverage estimation and dense cluster detection (Ma et al., 2024a; Xu et al., 2024), and instance segmentation particularly advantageous for separating overlapping fruits to ensure accurate single-fruit counting (Fang et al., 2025; Wang et al., 2025c). Regression and multi-modal fusion frameworks further integrate outputs from detection or segmentation with auxiliary parameters, such as fruit size, 3D canopy metrics, and environmental variables, enabling dimensional inversion, single-fruit mass estimation, and holistic yield prediction (Peng et al., 2025). Collectively, these methods offer complementary capabilities, balancing detection speed, fine-grained precision, and multi-source data integration to meet the diverse demands of modern orchard monitoring.

#### 5.2.1 Fruit counting via object detection

Object detection is the most widely used paradigm for direct fruit counting in orchard yield estimation. Its main objective is to localize, recognize, and quantify individual fruits using bounding boxes. Deep learning-based detection networks are broadly categorized into two types: two-stage and single-stage methods (Saleem et al., 2022). Two-stage networks, such as faster region-based convolutional neural network (Faster R-CNN), first generate region proposals and then classify and refine bounding boxes, offering high precision and strong adaptability to occlusion and varying fruit scales (Liu et al., 2025; Jiang et al., 2026a). Single-stage networks, such as the You Only Look Once (YOLO) series and Single Shot MultiBox Detector (SSD) (Liang et al., 2018; Liu et al., 2024a), integrate classification and regression in a single end-to-end framework, providing faster inference and suitability for real-time applications on mobile robots, autonomous rovers, and edge devices. Overall, two-stage approaches emphasize maximum precision, whereas single-stage methods balance speed and accuracy to meet both static and dynamic orchard monitoring needs.

Recent research on YOLO-based frameworks focuses on improving detection under complex orchard conditions through targeted enhancements:

- Multi-scale Representation: Multi-scale feature extraction improves the detection of small, dense, and scale-variable fruit targets. For apple fruitlet detection, a comparison of YOLOv8, YOLOv9, and YOLOv10 showed that YOLOv9 Gelan-e achieved the highest mAP@50 of 0.935, while YOLOv8n required only 4.1 ms per image, indicating a clear trade-off between accuracy and real-time performance (Sapkota et al., 2026).
- Feature Recalibration: Attention mechanisms help emphasize fruit-related regions and suppress background interference. YOLO11-ARAF, which integrates CARConv and the AFGCAM attention module, achieved 89.4% accuracy, 86.0% recall, 92.3% mAP@50 for apple detection in complex orchard scenes (Lin et al., 2025).
- Model Compression: Lightweight detectors reduce computational cost and support edge deployment. The Efficient Lightweight Detector achieved 87.4% detection accuracy with only 4.3×10^5^ parameters, 1.7 GFLOPs, and 156 FPS, showing strong potential for real-time orchard applications (Yang et al., 2024).
- Environmental Adaptability: Robust detectors are increasingly designed for illumination variation, occlusion, and complex backgrounds. Orchard-YOLO achieved 94.8% mAP@0.5 and approximately 25 FPS under simulated optical stress conditions, demonstrating improved stability for real-world orchard deployment (Wang et al., 2026).

These strategies collectively illustrate the shift from purely maximizing detection accuracy toward optimizing precision, speed, robustness, and deploy ability for real-world orchard applications.

#### 5.2.2 Multi-object tracking (MOT) algorithms

In video-based orchard yield estimation, repeated counting is a major challenge because the same fruits may appear in multiple consecutive frames during continuous image acquisition. This problem is especially common for mobile platforms operating in orchard rows under changing viewpoints, camera motion, and partial occlusion. Multi-object tracking (MOT) algorithms address this issue by associating the same fruit across frames, thereby reducing duplicate counting and improving the reliability of cumulative yield estimation (Jong et al., 2022; Villacrés et al., 2023). For example, AppleYOLO integrates an improved YOLOv8 detector with Deep OC-SORT for apple yield estimation, achieving 98.5% mAP50, with improvements of 1.0% and 5.1% over the original YOLOv8 baseline (Tan et al., 2025), respectively. This indicates that combining accurate detection with online tracking can effectively improve fruit counting stability in large orchard scenes.

Existing MOT methods in orchard monitoring mainly include tracking-by-detection and temporal modeling approaches. Tracking-by-detection methods associate detection results across frames using motion and appearance cues, and are widely adopted because of their simple structure and good real-time performance (Saraceni et al., 2024; Zhou et al., 2024). Temporal modeling methods further incorporate sequential or spatio-temporal information, which makes them more suitable for handling occlusion, viewpoint variation, and temporary fruit disappearance in complex orchard environments (Wu et al., 2025b). A recent dynamic Kalman filtering method combined an improved YOLO-based detector with a dynamically optimized Kalman filter and IoU–ReID association, achieving 95.0% MOTA for fruit tracking, while the final counting task reached R2 = 0.85 and RMSE = 1.57 (Zhai et al., 2025). In general, tracking-by-detection methods remain more practical for real-time deployment, whereas motion-adaptive and appearance-enhanced tracking methods provide stronger robustness in challenging orchard scenes.

#### 5.2.3 Semantic segmentation and instance segmentation

When fruits exhibit severe overlap or adhered boundaries, conventional object detection utilizing rigid bounding boxes frequently yields coarse localization and counting errors (Li et al., 2026). Semantic segmentation mitigates this by executing pixel-level classification (e.g., “fruit” vs. “non-fruit”), rendering it highly effective for canopy coverage estimation and dense cluster identification. Typical architectures—such as fully convolutional networks (FCNs), U-Net, and DeepLab—are often augmented with multi-scale atrous convolutions or skip connections to robustly extract features and generate spatial heatmaps. For instance, deploying DeepLabv3+ with ResNet18 and a circular Hough transform, Maheswari et al. (2025) accurately segmented mangoes to achieve a yield estimation R² of 0.98, underscoring the efficacy of semantic paradigms for macroscopic single-tree assessment.

However, pixel-level classification intrinsically lacks individual object distinction. Instance segmentation bridges this gap by jointly detecting targets and outputting pixel-level masks, outperforming semantic baselines in isolating adhered fruits within high-density canopies. While frameworks like Mask Region-based Convolutional Neural Network (Mask R-CNN) and You Only Look At Coefficients (YOLACT) have served as foundational baselines, recent literature highlights the superior real-time potential of YOLO-based instance segmentation. Sapkota et al. (2024) demonstrated this in apple orchards, achieving rapid inference (7.8–10.9 ms/image) and high precision (>0.90) for both green apple and branch segmentation. Furthermore, integrating transformer architectures can significantly enhance robustness against severe foliage occlusion; El Akrouchi et al. (2025) coupled MViTv2 with Cascade Mask R-CNN for tiny green citrus detection, achieving a full-tree counting R² of 0.81.

Beyond individual fruit quantification, instance segmentation facilitates advanced structural crop-load estimation. By utilizing a YOLOv8-based RGB-D system to segment trunks and branches, Ahmed et al. (2025) extracted limb cross-sectional areas to predict branch-level crop-load (RMSE = 3.95)—a critical parameter for robotic pruning and thinning decisions. Ultimately, these end-to-end paradigms are highly complementary: semantic segmentation excels in macroscopic region representation, while instance segmentation provides the fine-grained isolation and structural parsing requisite for complex orchard management.

Although deep learning models have achieved promising performance in fruit detection, segmentation, tracking, and yield regression, their practical deployment still faces several challenges. Model performance may be affected by dataset bias and domain shift caused by differences in cultivar, season, orchard structure, illumination, and imaging viewpoint. In addition, high-quality annotation remains labor-intensive, especially for segmentation tasks, while occlusion, shadows, and overlapping fruits may reduce model robustness. Therefore, future studies should pay more attention to model transferability, explainability, lightweight design, and real-time deployment on UAVs, UGVs, and embedded systems.

### 5.3 Methods based on UAV remote sensing

UAV-based remote sensing enables the collection of spectral, textural, and structural data for predicting orchard yield. Early models focused on vegetation indices (VIs) and canopy dimensions, but their accuracy was often limited by nonlinear orchard dynamics and physiological processes such as flowering and fruit abscission.

Recent advancements have integrated high-resolution orthomosaics and deep learning, making UAV-based methods central to yield estimation. For instance, Apolo-Apolo et al. (2020) applied Region-CNN to UAV-derived apple orthomosaics, achieving an R2 of 0.86 for fruit counting and 0.80 for yield prediction. Similarly, YOLO11n detected litchi panicles with an mAP@0.5 of 87.28% and a counting R2 of 0.9205, demonstrating the potential of floral metrics for pre-harvest modeling (Sun et al., 2025).

Studies increasingly combine UAV data with ground-based observations to enhance accuracy. Vijayakumar et al. (2023) fused UAV imagery with ground fruit counts, reducing citrus yield MAPE from 35.59% to 23.45%. Li et al. (2025a) combined UAV and Sentinel-2 data, with the best random forest model explaining 84% of apple yield variation. However, challenges remain in canopy occlusion, cross-regional generalization, and data acquisition costs.

### 5.4 Cross-method comparison and applicability analysis

This section aims to move beyond descriptive summarization by explicitly comparing the applicability boundaries of representative sensing and estimation pathways.

RGB imaging is widely used for fruit detection and counting due to its low cost and simple deployment, but it is sensitive to occlusion, illumination, and background interference. RGB-D and LiDAR add depth information, improving localization and canopy characterization, though with higher cost and computational demand. Hyperspectral and multispectral sensors support maturity and quality assessment but are less suitable for large-scale counting, while UAVs enable orchard-scale monitoring and spatial variability analysis, albeit with lower single-fruit precision.

Algorithmically, single-frame detection is efficient for static counting, whereas video-based multi-object tracking reduces repeated counts on mobile platforms but requires robust tracking under motion and occlusion. Instance segmentation effectively separates overlapping fruits, and regression or multi-modal fusion integrates multiple variables for tree- or orchard-level yield prediction. No single method is universally optimal; RGB/RGB-D suits high-precision tree-level estimation, while UAV and remote sensing support high-throughput orchard-scale monitoring. Future systems should focus on task-oriented integration, balancing accuracy, scalability, real-time performance, and deploy ability (as shown in **Table 4**).

**Table 4:** Cross-dimensional Applicability Matrix of Yield Estimation Methodologies.

Operational scale	Primary objective	Optimal sensor modality	Recommended algorithmic paradigm	Key engineering constraint
Single-Fruit/Local Branch	Rapid fruit counting & sizing	Ground RGB/RGB-D	Single-stage Detection (e.g., YOLO)/Instance Segmentation	Susceptibility to severe foliage occlusion and dynamic illumination.
Single-Tree/Dense Canopy	High-precision absolute counting & 3D profiling	Ground LiDAR/Stereo Vision	Multi-Object Tracking (MOT)/3D Point Cloud Processing	High computational overhead; complex field calibration
Single-Tree (Quality-focused)	Maturity assessment & internal trait evaluation	Portable Vis/NIR Spectrometers	Regression Models (PLS, SVR, RF)	High data dimensionality; limited to proximal, static operation.
Tree-Row/Plot Level	Continuous dynamic yield mapping	UGV + RGB/LiDAR/Hyperspectral	Video-based tracking (TBD)/Sensor Fusion	Intermittent occlusion during motion; real-time processing constraints
Whole-Orchard/Regional	Macroscopic yield forecasting & vigor zoning	UAV + Multispectral/RGB	Machine Learning Regression/Feature Fusion	Limited visibility of interior canopy fruits; restricted by aviation rule

## 6 What we know and what we need to learn

While the integration of advanced sensing and deep learning has profoundly advanced orchard yield monitoring, bridging the gap between academic prototypes and commercial deployment requires a critical reassessment of our current cognitive boundaries. This section critically evaluates what has been achieved, the fundamental knowledge gaps that persist, and the socio-economic pathways necessary for real-world adoption.

### 6.1 What we know: the current state of knowledge

Current literature unequivocally demonstrates that relying solely on 2D vision is insufficient for complex canopies. We know that introducing depth information (e.g., via RGB-D cameras or LiDAR) is mandatory for resolving severe occlusion and overlapping, effectively shifting the paradigm from flat fruit counting to 3D spatial localization and canopy volume profiling. Algorithmically, object detection and instance segmentation frameworks have reached a high level of maturity in static or controlled environments, significantly outperforming traditional hand-crafted feature extraction in mitigating illumination and background interference. Furthermore, tracking-by-detection methods in video streams have proven effective in reducing redundant counting on mobile platforms.

### 6.2 What we need to learn: unresolved challenges and research gaps

Despite these advancements, several critical knowledge gaps regarding absolute yield estimation remain largely unaddressed:

- From Phenotypic Counting to Biomass Inversion: Most existing models treat yield purely as a discrete counting problem (generating bounding boxes or masks). However, actual harvestable yield is a continuous mass variable. There remains a profound gap in accurately translating 2D projected areas or partial 3D point clouds into precise single-fruit weight and total biomass under severe canopy occlusion. Future research must pivot towards robust geometric reconstruction and mass inversion algorithms rather than stopping at fruit detection.
- Robust Multi-Object Tracking (MOT) in Highly Dynamic Canopies: While tracking algorithms reduce duplicate counts, they frequently fail during continuous UGV row-scanning when fruits temporarily disappear behind dense foliage and reappear across frames. We need to learn how to build more robust spatiotemporal memory into our models to maintain ID consistency over extended occlusions without accumulating severe cumulative counting errors.
- Current deep learning models suffer from severe domain shift. A counting model trained on a highly structured 2D V-trellis orchard typically fails when transferred to traditional 3D spherical canopies. The lack of universal, generalized feature representation for diverse, unstructured tree architectures remains a critical bottleneck.

### 6.3 Socio-economic feasibility and pathways to adoption

The ultimate bottleneck in intelligent yield monitoring is not merely algorithmic precision, but socio-economic feasibility—particularly in developing regions where financial and technical resources are acutely constrained. The prohibitive capital expenditure for high-end sensors and autonomous platforms currently restricts widespread adoption. Therefore, future research must prioritize cost-effective deployment, focusing on low-cost vision systems (e.g., consumer-grade RGB-D) and edge-computing paradigms that function reliably in offline, open-field environments without requiring complex infrastructure.

Furthermore, motivating farmer adoption depends fundamentally on demonstrating a clear economic return on investment (ROI) and ease of use. Systems must feature intuitive interfaces (e.g., smartphone-generated yield heatmaps) that directly translate raw data into actionable insights for precision thinning and harvest logistics. To overcome the digital divide and minimize training requirements, the industry should explore “Monitoring-as-a-Service” (MaaS) business models and policy-driven subsidies, enabling farmers to access cutting-edge yield mapping without intensive upfront capital or technical expertise.

## 7 Conclusion

Orchard yield monitoring has evolved from empirical field sampling and isolated image analysis toward an integrated intelligent perception paradigm that combines biological indicators, multi-source sensing, and data-driven modeling. This review shows that accurate yield estimation is not merely a fruit-counting problem, but a complex phenotyping task involving the joint characterization of fruit number, size, volume, spatial distribution, canopy structure, flowering intensity, and spectral maturity attributes. Direct fruit-level traits provide the most explicit basis for crop-load estimation, whereas indirect structural and physiological indicators extend the monitoring scale from individual fruits and trees to rows, plots, and whole orchards. Therefore, the future development of orchard yield monitoring should be understood as a shift from single-dimensional quantity estimation to multi-dimensional yield characterization.

Recent advances in RGB imaging, RGB-D sensing, LiDAR, hyperspectral/multispectral sensing, UAV remote sensing, and unmanned ground platforms have greatly expanded the technical boundaries of orchard yield monitoring. Meanwhile, deep learning methods, including object detection, instance segmentation, multi-object tracking, point-cloud analysis, remote-sensing regression, and multi-modal fusion, have substantially improved detection accuracy, counting stability, and yield prediction capacity under complex orchard conditions. However, the reviewed evidence also indicates that no single sensor, platform, or algorithm can independently overcome the inherent challenges of commercial orchards. Severe canopy occlusion, fruit overlap, variable illumination, heterogeneous tree architecture, and background interference continue to limit model robustness, while high computational cost, weak cross-environment generalization, and the lack of standardized benchmark datasets constrain large-scale deployment.

Future research must transcend isolated method-level optimization and pivot toward system-level intelligence and socio-economic viability. Priority should be given to occlusion-aware tracking, the accurate translation of fruit counts into precise biomass/weight estimates, and the development of lightweight, edge-deployable models that generalize across diverse canopy architectures. Crucially, the widespread adoption of these technologies—especially in resource-constrained and developing regions—depends on minimizing hardware costs, simplifying user interfaces, and establishing clear economic returns through optimized labor logistics and precision crop load management. By bridging the gap between advanced algorithmic perception and pragmatic, cost-effective agronomic decision-making, future systems will transform yield estimation from a retrospective counting exercise into an actionable, sustainable catalyst for the global modernization of fruit production.
